# Medial buttressing of the quadrilateral surface in acetabular and periprosthetic acetabular fractures

**DOI:** 10.1371/journal.pone.0243592

**Published:** 2020-12-07

**Authors:** Pol Maria Rommens, Michiel Herteleer, Kristin Handrich, Mehdi Boudissa, Daniel Wagner, Johannes Christof Hopf

**Affiliations:** Department of Orthopaedics and Traumatology, University Medical Center, Johannes Gutenberg-University Mainz, Mainz, Germany; University Hospital Zurich, SWITZERLAND

## Abstract

**Background:**

In geriatric acetabular fractures, the quadrilateral plate is often involved in the fracture pattern and medially displaced. Open reduction and internal fixation (ORIF) includes reduction of the quadrilateral plate and securing its position. In this study, the concept of medial buttressing in acute and periprosthetic acetabular fractures is evaluated.

**Materials and methods:**

Patients, who sustained an acetabular fracture between 2012 and 2018, in whom ORIF with a specific implant for medial buttressing was performed, were included in the study. Patients were divided in two groups; acute acetabular fractures (group 1) and periprosthetic acetabular fractures (group 2). Demographics, type of fracture, surgical approach, type of implant for medial buttressing, comorbidities, general and surgical in-hospital complications and length of hospital stay were recorded retrospectively. The following data were collected from the surviving patients by telephone interview: EQ-5D-5L, SF-8 physical and SF-8 mental before trauma and at follow-up, UCLA activity scale, Parker Mobility Score and Numeric Rating Scale.

**Results:**

Forty-six patients were included in this study, 30 males (65.2%) and 16 females (34.8%). Forty patients were included group 1 and six patients in group 2. The median age of patients of group 1 was 78 years. Among them, 82.5% presented with comorbidities. Their median length of in-hospital stay was 20.5 days. 57.5% of patients suffered from in-hospital complications. The concept of medial buttressing was successful in all but one patient. ORIF together with primary total hip arthroplasty (THA) was carried out as a single stage procedure in 3 patients. Secondary THA was performed in 5 additional patients (5/37 = 13.5%) within the observation period. Among surviving patients, 79.2% were evaluated after 3 years of follow-up. Quality of life, activity level and mobility dropped importantly and were lower than the values of a German reference population. SF-8 mental did not change. The median age of patients of group 2 was 79.5 years, all of them presented with one or several comorbidities. The median length of in-hospital stay was 18.5 days. 50% of patients suffered from in-hospital complications. The concept of medial buttressing was successful in all patients. 5 of 6 patients (83.3%) could be evaluated after a median of 136 weeks. In none of these patients, secondary surgery was necessary. Quality of life, activity level and mobility importantly dropped as well in this group. SF-8 mental remained unchanged.

**Conclusion:**

In geriatric acetabular fractures with involvement and medial displacement of the quadrilateral plate, medial buttressing as part of ORIF proved to be reliable. Only 13.5% of patients of group 1 needed a secondary THA within 3 years of follow-up, which is lower than in comparable studies. Despite successful surgery, quality of life, activity level and mobility dropped importantly in all patients. The loss of independence did however not influence SF-8 mental values.

## Introduction

Acetabular fractures are complex and incapacitating injuries. The column concept, which forms the basis of a universally accepted classification of acetabular fractures, has been introduced by Judet et al in the 1960’s [1]. Additional to the anterior and posterior column, the authors described the quadrilateral surface, which forms the inner wall of the acetabulum and is the lateral wall on both sides of the small pelvis [2].

The quadrilateral surface has come under increased attention due to the specific patterns of geriatric acetabular fractures [3]. The number of acetabular fractures in the elderly meanwhile overtops the number in younger persons [4, 5]. Older persons have a reduced bone quality and more often suffer low-energy trauma [6]. In geriatric acetabular fractures, subchondral impaction and fracture types involving the anterior column and the quadrilateral surface are frequent [7–9]. After a simple fall, the femoral head brakes and rotates the anterior column and the quadrilateral surface in opposite directions. The quadrilateral surface is dislocated towards medially with its posterior fracture line functioning as a hinge.

Open reduction and internal fixation (ORIF) of acetabular fractures in patients of old age is an accepted method of treatment [9, 10]. The goal is achieving an anatomical reduction and stable fixation of the acetabulum to prevent secondary osteoarthritis of the hip joint [11]. An alternative treatment is primary total hip arthroplasty (THA) [12, 13]. But there is an enhanced risk of symptomatic or asymptomatic cup loosening in THA after acetabular fracture [14]. ORIF of the acetabular fracture is rarely combined with primary THA. In this intervention, ORIF has the primary goal of creating a stable socket for cup placement [15]. The insertion of an additional or specific implant at the inner side of the reduced quadrilateral surface may be a good solution in counteracting the supero-medial forces to the hip joint and preventing secondary protrusion of the femoral head or loosening of the acetabular cup [16–18].

Letournel developed the ilioinguinal approach for open reduction and internal fixation of acetabular fractures with involvement of the anterior column and quadrilateral plate. Optimal indications for this approach are anterior wall, anterior column, anterior wall or column with posterior hemi-transverse and both column fracture [19]. The modified Stoppa and the pararectus approach are intrapelvic approaches, which have been developed more recently [20, 21]. Both enable a direct exposure of the lower part of the anterior column and the quadrilateral surface.

Until today, there is a lack of knowledge about optimal treatment of fractures of the acetabulum in patients of higher age. There are only a few studies with limited follow-up and there are no recommendations available. In this study, we analysed the efficacy of the concept of medial buttressing in ORIF of geriatric acetabular fractures or periprosthetic acetabular fractures.

## Patients and methods

We identified all patients with an acetabular fracture, treated by ORIF between 2012 and 2018 (7-year period) at the Department of Orthopaedics and Traumatology of the University Medical Centre Mainz. Patients, who had ORIF without an additional or specific implant for medial buttressing, were excluded (Table 1).

**Table 1:** Flowchart of excluded and included patients.

Selection of all patients from 2012 to 2018 presented in our Department with ICD code of S32.3, S32.4. S32.5, S32.7 or S32.8	
Number: 709	
Checking: coding of fracture of the acetabulum	
Yes	No: exclusion of 451 patients
Checking: patients with ORIF and implant for medial buttress	
Yes	No: exclusion of 212 patients
Patients with acetabular fractures receiving ORIF and medial buttress	
Number: 46	

Only patients with a medial buttress were included in our study (group 1). Patients with periprosthetic acetabular fractures were included as well, provided that they received a medial buttress as part of ORIF (group 2). Patients were included after informed consent and oral approval. All data were anonymized before analysis. The study was approved by the local ethics committee (Ethics Commission of the State Chamber of Medicine in Rhineland-Palatinate (Reference: 837.140.17 (10974))).

Demographic data, surgical approach, implant used for medial buttress, general and surgical postoperative complications were recorded in all patients. The fractures of patients belonging to group 1 were classified according to the Letournel-classification [1]. The quality of reduction of the fractures of group 1-patients was analyzed and classified according to the Matta-criteria by an independent observer (JH) [22].

Patients or their relatives were contacted by phone and asked to answer several questionnaires. If this was not possible, their general practitioner or the bureau of vital statistics were asked about vital status, registering date of death. Of all surviving patients, quality of life (QoL), mobility and independence were graded with the European Quality of Life 5 Dimensions 5 Level (EQ-5D-5L) questionnaire [23, 24], with the Short Form-8 (SF-8) [25] and with the UCLA activity score [26]. The pain level was rated with the Numeric Rating Scale (NRS) [27] and the mobility with the Parker Mobility Score [28]. During the same interview, the EQ-5D-5L, Short-Form 8 and Parker Mobility Score were also determined for the time prior to injury.

Mean values with standard deviation were given in case of normal distribution, median values with minimum and maximum together with 25 and 75 percentiles (IQR) in case of non-normal distribution. Continuous data were compared using the unpaired Mann-Whitney U test for non-normally distributed data. Linear regression was tested with Spearman Rho. The chi-square test was used to compare nominal groups. A p-value of ≤ 0.05 was considered statistically significant. The statistical analysis was done with SigmaStat (Systat Software GmbH, Erkrath, Germany).

## Results

During the study period, 258 patients with acetabular fractures were treated with ORIF in our Department. 212 patients were treated with ORIF without additional or specific implant for medial buttressing. Forty-six patients received a medial buttress with an additional or a specific implant as part of ORIF, and were included in our study. Table 1 shows a flowchart of excluded and included patients.

There were 30 males (65.2%) and 16 females (34.8%). Their median age was 78 years (range: 51–90 years, IQR: 72–82 years). Median body mass index (BMI) was 25.2 (range; 18.4–51.1, IQR: 23.2–27.0). Forty patients (87%) belonged to group 1 (ORIF of acute acetabular fracture), six patients (13%) to group 2 (periprosthetic acetabular fracture). The ilioinguinal approach was used in nearly three quarters of patients (74%). The different surgical approaches are depicted in Table 2.

**Table 2:** Approaches for ORIF with medial buttressing (n = 46).

	Number	**%**
Ilioinguinal appraoch	34	74.0
Modified Stoppa approach	9	20.0
Modified Stoppa approach with first window of Ilioinguinal approach	3	6.0

Different implants were used for medial buttressing, In the early years of the study, an implant additional to the pelvic brim plate was chosen for medial buttressing. In later years, preference was given to anatomical preshaped plates with two interconnected, orthogonal parts. The suprapectineal quadrilateral surface plate (Stryker, Freiburg, Germany) was the most frequently used (Table 3).

**Table 3:** Implants for medial buttressing (n = 46).

** **	**Number**	**%**
Suprapectineal quadrilateral surface plate (Stryker Co)	20	43.0
Quadrilateral surface plate (DePuy Synthes Co)	16	35.0
Infrapectineal quadrilateral surface plate (Stryker Co)	4	9.0
Omega Plate (Medin Co)	4	9.0
One third tubular plate (DePuy Synthes Co)	2	4.0

### Group 1, acetabular fractures (n = 40)

**Fig 1:**
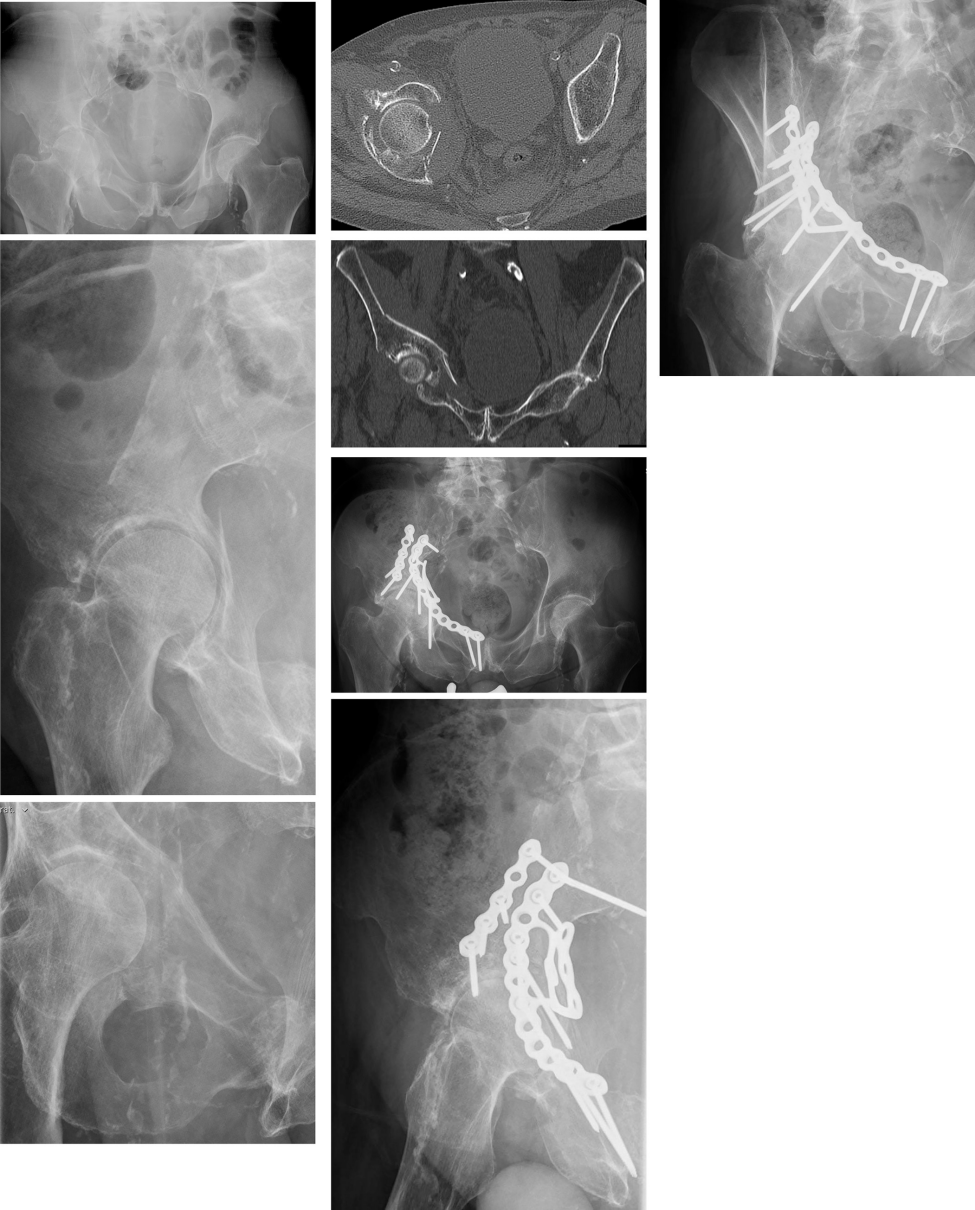
Sixty-eight year old man with anterior column and posterior hemitransverse fracture after alcoholic fall. (a) Pelvic ap overview. (b) Ala view. (c) Obturator view. (d) Axial CT cut through the acetabulum. The displaced quadrilateral plate is visible. (e) Coronal CT through the acetabulum. The quadrilateral plate is displaced medially togehter with femoral head. (f) Pelvicap overview one year after ORIF. The quadrilateral plate has been reduced and secured in its position with a suprapectineal quadrilateral surface plate. (g) Ala view. (h) Obturator view. The patient shows good mobility with a rolling walking frame.

There were 29 men (72.5%) and 11 women (27.5%) with a median age of 78 years (range: 51–90 years; IQR: 71–82 years). Median age of females (77 years) did not differ from that of males (78 years, p 0.905). Thirty-three patients (82.5%) presented with at least one comorbidity: cardiovascular diseases in 27, malignancies in 13, diabetes in 11, pulmonary diseases in 10, proven osteoporosis in 10, dementia in 6 and rheumatoid arthritis in 3. The most common fracture pattern was the anterior column with posterior hemi-transverse fracture, followed by both column fractures (Table 4) (Fig 1A–1H).

**Table 4:** Fracture types of acetabular fractures of patients in group 1 (n = 40).

** **	**Number**	**%**
Anterior column and posterior hemitransverse fracture	20	50
Both column fracture	15	37.5
Anterior column fracture	3	7.5
T-type fracture	2	5

The postoperative reduction was anatomic or perfect in 31 patients (78%) (Table 5).

**Table 5:** Quality of reduction in patients of group 1 (n = 40).

** **	**Number**	**%**
Anatomic (0–1 mm)	15	37.5
Perfect (1–2 mm)	16	40.0
Imperfect (2–3 mm)	8	20.0
Poor (> 3 mm)	1	2.5

Median length of stay was 20.5 days (range: 10–105 days; IQR: 14–28 days). Twenty-three of the 40 patients (57.5%) suffered one or several complications during the hospital stay. Pneumonia, urinary tract infection and surgical site infection were the most frequent (Table 6).

**Table 6:** General and surgical in-hospital complications in patients of group 1 (n = 40).

** **	**Number**	**%**
Pneumonia	9	22.5
Urinary tract infection	5	12.5
Surgical site infections	5	12.5
Pulmonary embolism	3	7.5
Skin ulcers	2	5.0
Gastrointestinal bleeding	1	2.5
Non-ST-segment elevation myocardial infarction (NSTEMI)	1	2.5
Recurrent hip dislocation	1	2.5
Iatrogenic bladder rupture	1	2.5

THA was performed in eight patients (20%), three (3/40 = 7.5%) during the primary operative procedure due to dome impression or femoral head damage deemed as non-reconstructable. Imperfect or poor reduction, secondary displacement of the quadrilateral surface, or posttraumatic osteoarthritis with immobilizing pain led to secondary THA in five patients (5/37 = 13.5%) after a median time interval of 36 weeks (range: 1–107 weeks). Three patients died during hospitalization. They were 78, 84 and 85 years old. Two patients died due to pneumonia and one due to multiple organ failure after surgical site infection. They died 15, 54 resp. 63 days after admission. Ten patients—including in-hospital deaths–died in the first year after trauma, resulting in a one-year mortality of 25%. They were older (median 80.5 years vs 77.0 years, p 0.050), had more often a complication during their stay at hospital (90% vs. 44%, p 0.013) but did not differ in the number of comorbidities (2.5 vs 1, p 0.113) or length of stay (median 26 days vs. 19 days, p 0.100). In total, 16 patients (40.0%) died before the interview could take place. They died at a median of 22 weeks (range: 2–200 weeks; IQR 7–127 weeks) after acetabular fracture, leaving 24 patients available for interview.

The interviews were performed in 19 of 24 (79.2%) patients at a median of 187 weeks (range: 53–334 weeks; IQR 75–273 weeks). 5 patients refused or were not available.

Median EQ-5D-5L index value dropped from 1.00 trauma to 0.51 at follow-up. The median SF-8 physical dropped from 55.40 to 30.53. The median SF-8 mental was 58.45 before the fractures and 57.72 at follow-up. Median PMS before the fractures was 9 and at follow-up 5. Median UCLA activity score was 3. Median NRS at rest, while sitting or on weight bearing was 0. Data with minimum and maximum as well as IQR is presented in Table 7.

**Table 7:** Group 1 (n = 40). 19 of 24 surviving patients (79.2%) were interviewed after a median time of 187 weeks.

	Median	Min	Max	25. Percentile	75. Percentile
EQ-5D index value before fractures	1.00	0.55	1.00	0.91	1.00
EQ-5D index value actual	0.51	-0.21	1.00	0.41	0.91
EQ-5D index value of representative German population	0.88				
SF-8 physical (PCS) before fractures	55.40	22.90	62.51	52.31	56.68
SF-8 physical (PCS) actual	30.53	19.46	58.00	25.96	46.23
SF-8 physical of representative German population	50.30				
SF-8 mental (MCS) before fractures	58.45	26.21	67.81	57.22	59.08
SF-8 mental (MCS) actual	57.72	17.86	67.32	49.91	60.90
SF-8 mental of representative German population	53.25				
PMS before fractures	9	6	9	9	9
PMS at follow-up	5	2	9	4	8
UCLA activity score	3	1	7	2	6
NRS at rest	0	0	5	0	0
NRS while sitting	0	0	4	0	0
NRS on weight bearing	0	0	10	0	3

### Group 2, periprosthetic acetabular fracture (n = 6)

**Fig 2:**
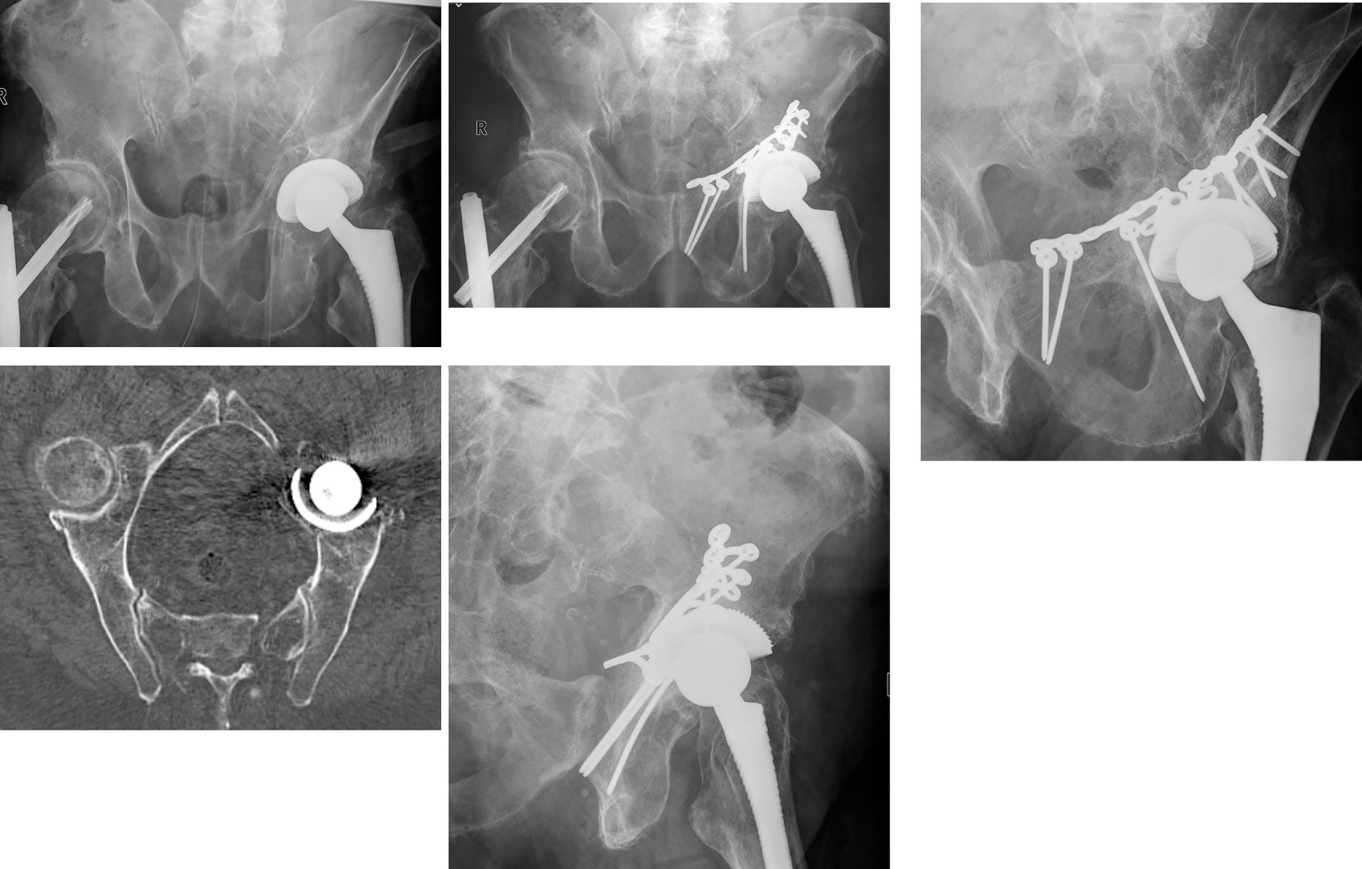
Seventy-eight old man with periprosthetic acetabular fracture after fall. The left hemiprosthesis has been inserted 9 years earlier. (a) Pelvic ap overview. (b) CT reconsruction at the level of the pelvic brim. The medial displacement of the quadrilateral plarte is clearly visible. (c) Pelvic overview 2 months after ORIF. The quadrilateral surface has been reduced and fixed with an Omega plate. (d) Ala view. (e) Obturator view. The patient shows good mobility with a rolling walking frame.

This group included 5 women and one man with a median age of 79.5 years (range: 76–87 years, IQR: 77.3–85.5 years). All patients presented with one or several comorbidities: cardiovascular diseases in five patients, diabetes, dementia and proven osteoporosis in three patients, pulmonary disease, malignancy and rheumatoid arthritis in one patient. In two patients with bipolar hip prosthesis, an ORIF with medial buttress was performed (Fig 2A–2E). Two patients underwent ORIF with medial buttress and exchange of acetabular cup in a single stage session. Two patients had exchange of the cup in a second procedure 6 and 18 weeks after ORIF. Three patients suffered general complications: urinary tract infection, pneumonia or deep venous thrombosis each one. The median length of hospital stay was 18.5 days (14–28 days; IQR: 14–25 days). There was no in-hospital mortality in this group.

At follow-up, one patient had died 82 weeks after fracture, her age was 83.2 years. Five surviving patients could be evaluated after a median time of 136 weeks (45–210 weeks).

Median EQ-5D-5L index value of the 5 patients dropped from 0.75 to 0.18.]. The median SF-8 physical dropped from 44.32 to 27.98. The median SF-8 mental before the fractures was 37.65 and at follow-up 40.89. Median PMS before the fractures was 4, at follow-up 3. Median UCLA activity score was 1.6. Median NRS of patients at rest was 3, while sitting 3 and on weight-bearing 6.5. Data with minimum and maximum as well as IQR is presented in Table 8.

**Table 8:** Group 2 (n = 6).

	Median	Min	Max	25. Percentile	75. Percentile
EQ-5D index value before fractures	0.75	0.26	0.83	0.52	0.79
EQ-5D index value actual	0.18	0.13	0.49	0.18	0.28
SF-8 physical (PCS) before fractures	44.32	22.95	53,39	27.10	46.23
SF-8 physical (PCS) actual	27.98	21.50	33.32	23.39	31.55
SF-8 mental (MCS) before fractures	37.65	31.15	61.45	33.41	58.86
SF-8 mental (MCS) actual	40.89	17.71	45.99	30.82	42.02
PMS before fractures	4	1	9	4	8
PMS at follow-up	3	0	3	0	3
UCLA activity score	1.6	1	2	1	2
NRS at rest	3	0	6	2	5
NRS while sitting	3	0	8	2	6
NRS on weight bearing	6.5	0	10	3	7

All 5 surviving patients were interviewed after a median time of 136 weeks.

## Discussion

Our study cohort represents a unique geriatric population with a mean age of 78 years, As in other studies, we found a predominance of men in geriatric acetabular fractures, whereas the incidence of women is much higher in fragility fractures of the pelvis [9, 29]. In group 1, 82.5% of patients presented with at least one comorbidity and more than half of them suffered a complication during hospital stay. This data impressively shows the high risks, to which these patients are exposed, once they suffer a major fracture. Only five patients (13.5%) needed a THA during an observation period of more than 3 years, which is considered enough to detect most cases of osteoarthritis after acetabular fractures [30]. This low rate underlines the efficacy of medial buttressing, provided that the reconstruction of the acetabulum was anatomic or nearly anatomic. In group 2, all patients presented with comorbidities, half of them suffered a complication during hospital stay. There were no surgical complications. There was no secondary dislocation after ORIF with medial buttressing. Secondary surgery was not needed during the observation period. This data underlines the efficacy of medial buttressing in patients with periprosthetic acetabular fractures.

The force direction of the femoral head is decisive for the emerging pattern of acetabular fractures. When the hip joint is in extension during the fall, there is an increased occurrence of anterior wall and anterior column fractures [31]. This is the typical fracture pattern of elderly patients, who suffered a low-energy trauma [3, 9]. Subchondral impaction and displacement of the quadrilateral plate with medial protrusion of the femoral head are frequent concomitant findings [32].

Conservative treatment of these fractures is only acceptable in patients with non-displaced fractures or high surgical risks [33, 34]. With ORIF, the quadrilateral surface is addressed as part of the reduction process. Non-anatomical reduction and articular incongruence lead to early secondary osteoarthritis [35]. In our experience, also primary anatomical reposition and fixation led to secondary medial displacement using suprapectineal plating without medial buttressing. Securing the quadrilateral plate in its reduced position is best obtained through a medial stabilizer [17]. Medial buttressing is of special importance in elderly persons, who have a decreased bone mineral density and diminished holding power of screws.

The superior biomechanical properties of medial buttress-implants have been proven in several studies [35, 36]. In a recent paper, Chen et al favorize the use of suprapectineal plates above infrapectineal plates [37]. We used the one-third tubular plate twice and the construct failed once. The one-third tubular plate is too weak for securing the position of the reduced quadrilateral surface. Novel implants have two orthogonal parts that are interconnected in a very stiff manner. Suprapectineal implants were used in 39 patients, infrapectineal in 5. The Omega plate, which was used 4 times, can be regarded as a hybrid implant, because the insertion of suprapectineal and infrapectineal screws is possible. There were no secondary dislocations with these novel implants.

In comparison with recent studies, we found a low rate of secondary THA in our series. Carroll et al. reviewed 93 patients with a mean age of 67 years and a follow-up of five years. The rate of secondary THA was 31% [38]. Jeffcoat et al. presented 41 patients with a mean age of 67 years and with a minimum of two years follow-up. Conversion to THA was needed in 27% [11]. The question remains if THA should be performed in the same operative procedure as ORIF or delayed. Rickman et al presented 12 patients, in which a combination of ORIF and THA was performed in the same operation. No cup migration was seen after a mean time of 18 months [39]. Borg et al performed ORIF with THA in one operative session in 13 patients. No patient needed secondary surgery within three years of follow-up [40]. Sermon et al reviewed 121 patients with THA after an acetabular fracture. The revision rate after secondary THA was 22%. This rate was significantly higher than the revision rate of 8% in the early THA group [41]. The median age of our patients was higher than in all abovementioned series and the conversion rate was lower. Our data suggest that ORIF with medial buttressing may successfully protect the reconstructed hip joint from secondary displacement with need of THA.

The EQ-5D-index value of all patients dropped far below that of a representative German population, which is 0.88 (SD 0.18) [42]. SF-8 physical also dropped below the value of a representative German population, which is 50.30 (SD: 8.39). On the contrary, the values of SF-8 mental did not drop and were similar to those of a representative German population, which is 53.25 (SD: 7.82) [25]. In group 1, a PMS of 5 and an UCLA activity scale of 3 means that patients only participate in light physical activities. There was no pain at any activity. Data on quality of life, level of independence and mobility is available from another study on ORIF of acetabular fractures in patients of old age, performed in our institution [9]. In group 2, a PMS of 3 and an UCLA activity scale of 1.6 means that those patients hardly participated at any physical activity. Pain level was moderate to high depending on the degree of mobilisation.

This study has several limitations. Due to its retrospective design, not all data of the patients could be collected. The study group is small and some patients were lost to follow-up. Nevertheless, 80% of surviving patients of group 1 and 83% of group 2 were available for evaluation. Estimation of quality of life, level of independence and mobility was performed with telephone calls instead of physical examinations. Multicentre, prospective observational studies with larger patient cohorts can bring more clarity in the question of optimal treatment of acetabular fractures with medial displacement of the quadrilateral plate in patients of old age.

## Conclusion

Medial buttressing of the quadrilateral surface as part of ORIF of acetabular fractures in geriatric patients leads to a stable acetabular cavity. Due to their high age and comorbidities, patients are at high risk of in-hospital complications. The treatment concept allowed securing the hip joint without the need of conversion to THA in the vast majority of patients with acute acetabular fractures. In patients with periprosthetic acetabular fractures, ORIF with medial buttressing efficiently restores stability. Despite successful surgical treatment; quality of life, activity level and mobility importantly dropped in these patients of old age.

## Supporting information

S1 File (XLSX)
